# Effect of an Educational Program on Nurses' Knowledge and Practice of Oxygen Therapy

**DOI:** 10.7759/cureus.39248

**Published:** 2023-05-19

**Authors:** Ghulam Mustafa

**Affiliations:** 1 Pediatric Medicine, College of Medicine-Shaqra University, Shaqra, SAU

**Keywords:** knowledge level, nurses, practice, oxygen therapy, educational program

## Abstract

Introduction: Past studies have shown that healthcare professionals may lack awareness and knowledge regarding oxygen therapy, and its implementation often has several obstacles. This study was carried out to investigate the effect of an educational program about oxygen therapy on nurses' knowledge and practices.

Methods: This cross-sectional, quasi-experimental study was conducted in 2022 at the pediatric department of Nishtar Hospital, Multan, where 160 nurses from primary and secondary health centers attended an educational program delivered in the pediatric department. The pre-test-post-test approach was used to evaluate the effectiveness of the structured educational program. The independent variable was the educational program, and the dependent variable was the nurses’ knowledge and practice about oxygen toxicity. Data analysis was performed using SPSS version 23 (IBM Corp., New York, USA). The data were tabulated as means and standard deviations for numerical values and frequency percentages for categorical values. The student's *t*-test and the chi-square test were applied to investigate any associations among variables.

Results: The average test scores before and after the implementation of the educational program were 10.75±2.65 and 17.52±2.04, respectively. The average post-test score was greater than that of the pre-test, and the difference was statistically significant (p<0.001).

Conclusion: The study found that after the implementation of the educational program, the knowledge and practices of nurses regarding oxygen therapy improved significantly, with the majority showing a positive attitude toward the program.

## Introduction

Oxygen therapy (OT) is a vital form of medical treatment in many different scenarios and is one of the primary treatments for patients with chronic respiratory distress [1]. Despite the significance of oxygen therapy, past studies have suggested that there may be a need for more awareness and knowledge among healthcare professionals concerning oxygen therapy and several obstacles in its implementation [2].

The use of oxygen as a therapeutic drug in critically ill patients has been in practice for many decades, regardless of the environment in which it was delivered [3]. The calculated OT required amount for patients with hypoxemia is often underestimated, and incorrect calculations may lead to fatal conditions [4]. In critically ill patients, oxygen should be administered safely and appropriately, which depends on a complete understanding of the purpose and benefits of its delivery method. The most common harmful effect due to oxygen delivery is the toxicity that can occur in cases where oxygen is delivered at a concentration above 50% for more than 24 hours [5].

Mainly the eyes, respiratory system, and central nervous system are affected by oxygen toxicity in the human body, with high-risk populations including deep-sea divers, premature infants, hyperbaric operation theater patients, and patients exposed to high levels of oxygen [6]. Therefore, efforts should be made to warn against the toxic effects of OT under continuous use. Furthermore, pulse oximetry and arterial blood gases (ABGs) should be monitored continuously, as harmful pulmonary changes can be irreversible [7].

Nurses should routinely inspect the mucous membrane and skin of the mouth to assess signs of any physical damage due to the tubing or oxygen toxicity [8]. This inspection could involve the detection of color changes, inflammation, ulceration, secretions, and other potential issues. It is essential to detect problems early, as this can prevent further complications and ensure that necessary treatments are given promptly. Regular inspections are critical for achieving optimal health and well-being [9].

It is evident that consistent educational programs held on a yearly basis can have a beneficial influence on the knowledge and execution of OT among healthcare practitioners [10]. By providing the necessary information and training, these programs can effectively aid physicians and nurses in the proper implementation of OT, subsequently leading to enhanced patient care and outcomes [11]. As a result, the objective of our study is to assess the effect of an educational program on the practices and knowledge of OT among nurses in our region.

## Materials and methods

The program was delivered in the emergency department of the hospital’s pediatric unit. The program’s primary objective was to improve nurses’ knowledge regarding OT, and the secondary objective was to enhance their understanding and description of the anatomy of the respiratory system. This work used a cross-sectional quasi-experimental research design with a pre-test-post-test approach to evaluate the effectiveness of a structured educational program.

The development of the educational program and the assessment tool used in this study resulted from the researcher's review of related literature [1,8,9] to assess nurses' knowledge regarding oxygen toxicity. The two-part tool was created to provide nurses with standardized educational information and their assessment process to ensure accuracy and consistency.

The first part of the tool consisted of a questionnaire. This questionnaire was designed to assess nurses’ knowledge of how to properly evaluate the patient’s oxygen toxicity levels, including their signs, symptoms, risk factors, and treatments. This part also focused on nurse demographics such as age, sex, workplace, qualification, and experience. All nurses were administered a validated pre-test questionnaire before starting the educational program. The questionnaire consisted of 20 questions and took approximately 15-20 minutes to complete it. Knowledge assessment was done two times through the same questionnaire: once at the beginning of the study (also named the pre-test assessment) and again immediately after the implementation of the educational program (also named the post-test assessment). 

After pre-test completion, the educational program was delivered, which involved several vital protocols, including proper hand hygiene, equipment provision, patient verification, respiratory assessment, and patient response evaluation. It also involved the correct technique of administering OT, considering age variations to adjust oxygen flow rates, placing the patient in a comfortable position after administering OT, taking safety precautions when leaving the room, documenting patient findings and essentially reporting any areas of concern. The program educated the nurses about the function of the respiratory system, the definition of oxygenation, indications of OT, oxygen humidification, interpretation of pulse oximetry, OT at home, patients' health education, and the nurses' role in oxygen delivery or toxicity.

The second part of the tool was a scoring system that used the responses to the questionnaire to assign a numeric value to the nurses' education level. In this tool, a correct answer is scored as “1,” and an incorrect answer is scored as “0”. The maximum total score is 20 and is categorized as either unsatisfactory (score below 12, i.e., 60% of the total) or satisfactory (score above 12).

The Institutional Review Board of the Institute of Mother and Child Care (I-MACCA) Multan, Pakistan, approved the research proposal with letter number CR/0622/0005 dated 12.06.2022. Written consent was obtained from nurses willing to participate in the study after the researcher explained its nature and purpose. The researcher also assured the nurses that the collected data would only be used for research purposes. Additionally, the nurses were assured of their confidentiality and anonymity and the right to refuse or withdraw from the study at any time and for any reason.

The data collected from the pre-test and post-test were analyzed to determine the effectiveness of the educational program. Descriptive and inferential statistical methods were used to find the differences between the pre-test and post-test scores. Data analysis was performed using SPSS version 23 ((IBM Corp., New York, USA). The independent variable was the educational program, and the dependent variables were the nurses’ knowledge and their intended practices about oxygen toxicity. The data were tabulated as means and standard deviations for numerical values and frequency percentages for categorical values. The student's t-test and the chi-squared test were applied to determine any association among variables.

## Results

Overall, 160 nurses were included in our study, of which 46 (28.8%) were charge nurses and 114 (71.3%) were staff nurses. The mean test scores and the significance of each question before and after the educational program are shown in Table 1. The differences in pre-test and post-test scores for questions eight, 12, 13, and 14 were found to be statistically significant (i.e., p<0.050).

**Table 1:** The average test score of each question before and after the implementation of the educational program. S: significant, NS: non-significant.

Question	Pre-test score	Post-test score	p-value	Decision
1	0.68±0.46	0.69±0.46	0.905	NS
2	0.74±0.43	0.67±0.47	0.083	NS
3	0.70±0.46	0.63±0.48	0.116	NS
4	0.81±0.39	0.81±0.39	1.000	NS
5	0.65±0.48	0.70±0.46	0.319	NS
6	0.44±0.49	0.51±0.50	0.219	NS
7	0.27±0.44	0.33±0.47	0.182	NS
8	0.46±0.50	0.59±0.49	0.004	S
9	0.68±0.46	0.71±0.45	0.458	NS
10	0.60±0.49	0.67±0.47	0.217	NS
11	0.40±0.49	0.44±0.49	0.356	NS
12	0.44±0.49	0.60±0.49	0.001	S
13	0.47±0.50	0.66±0.47	<0.001	S
14	0.66±0.47	0.77±0.42	0.042	S
15	0.58±0.49	0.61±0.49	0.613	NS
16	0.49±0.50	0.43±0.49	0.182	NS
17	0.36±0.48	0.39±0.49	0.458	NS
18	0.61±0.48	0.62±0.48	0.769	NS
19	0.67±0.47	0.73±0.44	0.114	NS
20	0.62±0.48	0.57±0.49	0.303	NS

The overall average test scores before and after the implementation of the educational program were 10.75±2.65 and 17.52±2.04, respectively. The average post-test score was greater than that of the pre-test, and the difference was statistically significant (p<0.001). Furthermore, the differences in the mean test score of staff nurses and charge nurses both before and after the implementation of the educational program were statistically significant (p<0.001), as shown in Table 2.

**Table 2:** The average test score of the nurses before and after the implementation of the educational program.

Category	N	Pre-test score	Post-test score	p-value
Overall	160	10.75±2.65	17.52±2.04	<0.001
Staff nurses	114	10.78±2.32	18.03±1.78	<0.001
Charge nurses	46	10.65±3.34	16.23±2.08	<0.001

**Figure 1:**
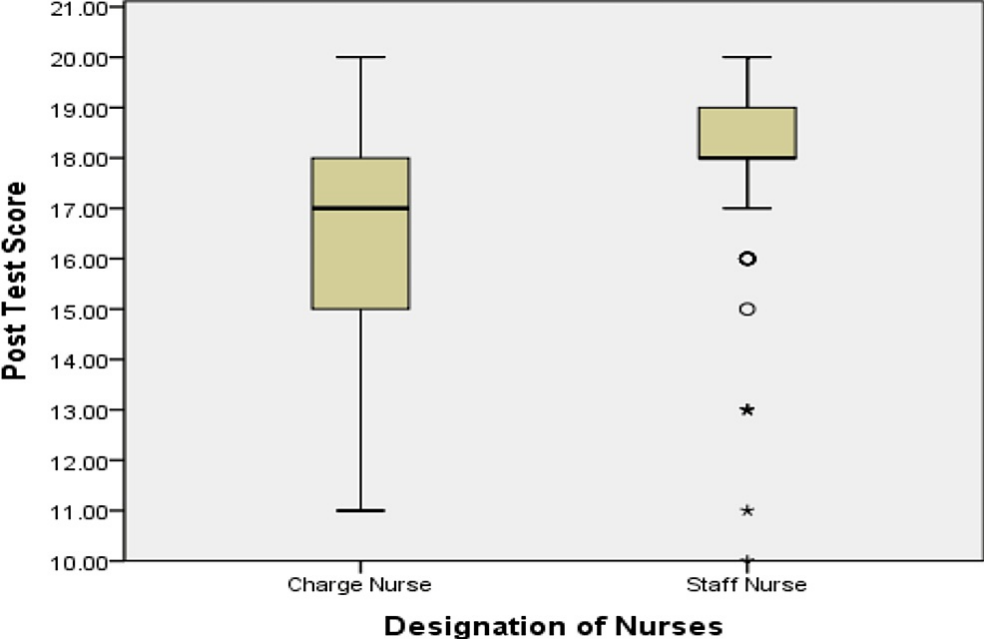
Relative improvement in the knowledge and practice of the charge nurses versus staff nurses after the educational program.

The average post-test scores of staff and charge nurses were 18.03±1.78 and 16.23±2.08, respectively. Therefore, the average post-test score of staff nurses was greater than that of charge nurses, and the difference was statistically significant (p<0.001), as shown in Figure 1.

## Discussion

Oxygen therapy is a necessary treatment for various medical conditions, and nurses play an essential role in assessing its need and ways of administering it [12]. However, there needs to be more evidence that nurses are provided with a regular annual training program to inform and support nursing practices related to OT. To address this knowledge gap, research is needed to better understand the needs of OT patients, identify best practices for the successful management of OT, and develop evidence-based guidelines for nurses to follow when providing OT [13]. Our study is the first in our region to explore the effects of such an educational program.

In our study, in parallel with our healthcare settings, all nurse participants were female, though the faculty has recently recognized male nursing as well. The majority of participants had nursing experience totaling 5-10 years. In a study conducted in 2012, Eastwood et al. [14] found that most nurses employed in emergency and intensive care units had bachelor’s degrees in nursing. Lack of participation in any previous annual training program may be because hospitals usually do not have a staff development program related explicitly to oxygen toxicity. This result is supported by Rochester [15], who found that nurses working in intensive care units need additional education (like training courses and refresher courses) to provide the best possible care to patients receiving OT and prevent oxygen toxicity.

Chen et al. [16] conducted a study in 2018 and found that most nurses surveyed had never attended any training course on OT, highlighting the need for more comprehensive education programs to tackle this and other healthcare-related issues. Such programs should include detailed information on how to safely and effectively administer OT and the most up-to-date guidelines about medical protocols and standards of practice. As demonstrated in a 2016 study conducted by Markocic et al. [17], understanding the potential for oxygen toxicity is critical for nurses to properly assess and identify any issues that could arise from OT. 

Healthcare professionals, such as nurses, are critical in administering OT to patients in critical situations. As Aloushan et al. [1] pointed out in 2019, a lack of knowledge can negatively impact patients' health outcomes. As such, nurses must be given adequate education to ensure they can adequately assess and administer OT safely and effectively. 

According to Urden et al. [18], nursing professionals’ knowledge of OT is often only moderate. To ensure that these professionals are adequately trained, in-service educational programs are needed to improve their knowledge and practice. These programs should focus on improving nurses’ understanding of OT, including oxygen delivery systems, oxygen saturation monitoring, and patient assessment; they should also cover the importance of OT in treating patients with respiratory conditions such as chronic obstructive pulmonary disease (COPD) and pneumonia, as well as OT in premature infants [19]. Furthermore, nurses should be taught how to recognize and manage the potential risks associated with OT, such as hypoxemia and hyperoxemia. Through these programs, nurses can understand OT comprehensively, enabling them to provide the best possible care to their patients [20].

The study underscores the need for a regular annual training program for nurses that should assess their knowledge and practices regularly. This assessment process can help identify areas of improvement or gaps in the nurses’ knowledge and performance while guiding further training or remedial actions to ensure they are well-equipped to provide the best patient care. More longitudinal research to check if such programs result in long-lasting effects is really necessary.

One of the primary limitations of this study focusing on the effect of an educational program on nurses' knowledge and practice of oxygen therapy was using a single methodology. The study exclusively relied on self-reported surveys to measure the effectiveness of the educational program, like Temsah et al.'s study [21], but it may be subject to recall bias and social desirability bias. Moreover, the study also lacked a control group, which made it difficult to discern whether any changes in knowledge or practice genuinely resulted from the educational program or other factors. Additionally, the study's sample size could have been more extensive, which may reduce the generalizability of the findings. Finally, the study was conducted in a single clinical setting, which may not represent the broader range of practice contexts in which OT is administered, thereby limiting the generalizability of the study's findings to other settings.

## Conclusions

Implementing an educational program on OT showed significant direct improvements in the knowledge and intended practices of nurses. The program focused on providing nurses with up-to-date information on the correct use of OT and the importance of monitoring its administration. The results demonstrated that a structured educational program can enhance nurses’ competence and confidence when administering OT, improving patient care and outcomes. It is essential for healthcare facilities to prioritize continuing education opportunities for nurses to ensure that they are equipped with the knowledge and skills necessary to provide optimal patient care.

## References

[1] Aloushan AF, Almoaiqel FA, Alghamdi RN, Alnahari FI, Aldosari AF, Masud N, Aljerian NA (2019). Assessment of knowledge, attitude and practice regarding oxygen therapy at emergency departments in Riyadh in 2017: a cross-sectional study. World J Emerg Med.

[2] Kinsman L, Cooper S, Champion R (2021). The impact of web-based and face-to-face simulation education programs on nurses' response to patient deterioration: a multi-site interrupted time series study. Nurse Educ Today.

[3] Bunkenborg G, Bundgaard K (2019). A mixed methods exploration of intensive care unit nurses' perception of handling oxygen therapy to critically ill patients. Intensive Crit Care Nurs.

[4] Siwach I, Yadav SR, Kumar R (2022). A knowledge, attitude and practice study of prescribing oxygen amongst interns in a tertiary care hospital. Eastern Ukrainian Med J.

[5] Foley C, Dowling M (2019). How do nurses use the early warning score in their practice? A case study from an acute medical unit. J Clin Nurse.

[6] Kim SO, Choi YJ (2019). Nursing competency and educational needs for clinical practice of Korean nurses. Nurse Edu Pract.

[7] Graham H, Bakare AA, Fashanu C (2020). Oxygen therapy for children: a key tool in reducing deaths from pneumonia. Pediatr Pulmonol.

[8] Lee W, Kim M, Kang Y (2020). Nursing and medical students’ perceptions of an interprofessional simulation-based education: a qualitative descriptive study. Korean J Med Edu.

[9] Desalu OO, Ojuawo OB, Adeoti AO (2022). Doctors’ and nurses’ knowledge and perceived barriers regarding acute oxygen therapy in a tertiary care hospital in Nigeria. Adv Med Educ Pract.

[10] Joosten SA, Koh MS, Bu X (2007). The effects of oxygen therapy in patients presenting to an emergency department with exacerbation of chronic obstructive pulmonary disease. Med J Aust.

[11] Austin MA, Willis KE, Blizzard L (2010). Effect of high flow oxygen on mortality in chronic obstructive pulmonary disease patients in prehospital setting: randomised controlled trial. BMJ.

[12] Aubier M, Murciano D, Milic-Emili J (1980). Effects of the administration of O2 on ventilation and blood gases in patients with chronic obstructive pulmonary disease during acute respiratory failure. Am Rev Respir Dis.

[13] Henrichs KA, Makic MB (2021). A quality improvement project to increase oxygen therapy adherence in patients newly prescribed oxygen at discharge. Med Surg Nurs.

[14] Eastwood G, Reade M, Peck L (2012). Critical care nurses’ opinion and self-reported practice of oxygen therapy: a survey. Aust Crit Care.

[15] Rochester H (2017). Seventh report of the joint national committee on prevention, detection, evaluation, and treatment of high blood pressure. Treat Health Care J.

[16] Chen Y, Niu M, Zhang X (2018). Effects of home‐based lower limb resistance training on muscle strength and functional status in stable chronic obstructive pulmonary disease patients. J Clin Nurs.

[17] Markocic S, Humphries M, Tarne K (2016). What are the risks and knowledge deficits for prescribing and administering opioids in the ward environment? A quality project on assessing and improving knowledge. Nurse Edu Pract.

[18] Urden LD, Lough ME, Kathleen MS (1996). Priorities in critical care nursing, 2nd edition. Dimensions Crit Care Nurs.

[19] Wang T, Tan JY, Xiao LD (2017). Effectiveness of disease-specific self-management education on health outcomes in patients with chronic obstructive pulmonary disease: an updated systematic review and meta-analysis. Patient Edu Counseling.

[20] Vargas F, Saint-Leger M, Boyer A (2015). Physiologic effects of high-flow nasal cannula oxygen in critical care subjects. Respir Care.

[21] Temsah MH, Aljamaan F, Alhaboob A (2022). Enhancing parental knowledge of childhood and adolescencesafety: an interventional educational campaign. Medicine.

